# Geriatrics Evaluation and Management in the Veterans Administration—An Historical Perspective

**DOI:** 10.3390/geriatrics3040084

**Published:** 2018-11-25

**Authors:** James S. Powers, Kathryn J. Eubank

**Affiliations:** 1 The Tennessee Valley Healthcare System Geriatrics Research, Education, and Clinical Center, Vanderbilt University School of Medicine, 7159 Vanderbilt Medical Center East, Nashville, TN 37232, USA; 2 The San Francisco VA Medical Center, The University of California San Francisco School of Medicine, San Francisco, CA 94121, USA; kathryn.eubank@ucsf.edu

**Keywords:** geriatric evaluation and management, Acute Care for Elders, geriatric rehabilitation

## Abstract

Comprehensive geriatric assessment, defined as an interdisciplinary assessment and development of an overall plan of treatment and follow-up, has become a fundamental part of clinical geriatric care. Since the 1970s, the US Department of Veterans Affairs (VA) has encouraged the development of geriatric evaluation and management programs. Evolution of geriatric evaluation and management has occurred over time and many VA medical centers have transferred inpatient geriatric evaluation programs to long-term care Community Living Centers, home, and outpatient settings. Availability of geriatric resources and trained personnel across the continuum of care as well as administrative collaboration between care components are critical to the successful implementation of geriatric services. Facilities may need to prioritize their resources and utilize the most effective and relevant elements of geriatric evaluation and management according to patient population needs, available space, resources, and institutional priorities. New risk assessment tools derived from the VA’s experience in geriatric evaluation may be useful for targeting services for other high-risk populations.

## 1. Introduction

Comprehensive geriatric assessment, defined as an interdisciplinary assessment and development of an overall plan of treatment and follow-up, has become a fundamental part of clinical geriatric care. This concept originally focused on inpatient populations. Since the 1970s, the Veterans Administration (VA), which provides patient care and federal benefits to veterans and their dependents, has encouraged the development of Geriatric Evaluation and Management (GEM) programs, with at least 105 inpatient GEM programs reported in 1993 [1]. Standard GEM programs consist of (1) an interdisciplinary core team composition including a physician, nurse, and social worker (with additional personnel as appropriate for individual patients i.e., dietitian, therapist, pharmacist, psychologist), (2) comprehensive assessment, (3) selective admissions for patients with potential to benefit from GEM, (4) location in acute or long-term care location, and (5) provision for inpatient rehabilitation and follow-up [1]. Nonstandard units lack one or more of these characteristics. Inpatient geriatric units are associated with improved diagnostic accuracy and management of geriatric syndromes, reduced numbers of medications, improved functional outcome, fewer long-term care admissions, and reduced acute care hospital readmissions, improved medication utilization, and establishment of patient-centered goals of care [2,3,4]. A 1993 VA GEM program assessment revealed the average GEM unit then consisted of a mean 12.7 beds (standard unit) to 15.4 beds (non-standard unit) size, with a mean length of stay (LOS) of 24.5 days (standard unit) to 69.9 days (non-standard unit) [1]. Since that time other geriatric evaluation models have been developed. Acute Care for Elders (ACE) units are specially designed inpatient units located in the acute care setting and focused on maintaining function, early discharge planning, and patient-centered age-appropriate care [2,4]. A meta-analysis of ACE programs published in 2012 showed that ACE units, compared to usual hospital care, had significantly lower length of stays, lower hospital costs, and more discharges home. In addition, the ACE model was associated with fewer falls, less delirium, and less functional decline [5]. Mobile ACE units (MACE), acute care geriatric services without a geographic bed location, have since been shown to have similar outcomes [6].

The VA requires availability of geriatric evaluation services for all military veterans as mandated in the 1999 Veterans Millennium Healthcare and Benefits Act (Mill Bill). No longer is geriatric evaluation and management confined to inpatient units. These services may currently be located in inpatient, long-term care-community living center (CLC) or outpatient settings [7]. Geriatric evaluation consists of a comprehensive, multi-dimensional assessment and development of an interdisciplinary plan of care undertaken by an interdisciplinary team of healthcare professionals for a target a group of clinically defined high-risk high-need patients who are most likely to benefit from these services. Over time, geriatric evaluation has been applied to other health care settings including home, long-term care, and outpatient locations. Many VA medical centers have developed a full spectrum of geriatric services and transferred inpatient geriatric evaluation programs to these settings. The VA experience in geriatric program development may guide non-VA systems in developing health services for their high-risk populations. We track the evolution of GEM services at Tennessee Valley Healthcare System which transferred its Geriatric Evaluation Unit to the long-term care-community living center in November 2017, and contrast the Tennessee Valley Healthcare System experience with GEM to the ACE Unit at the San Francisco VA Medical Center discussing the evolution of geriatric evaluation services in the VA from a 30-year clinical, operations and policy perspective.

## 2. Program Description 

### 2.1. Setting

A GEM unit was initiated in 1987 at the Nashville Campus of the Tennessee Valley Healthcare System as an intermediate care unit, a specially designed inpatient unit with an average length of stay less than 30 days, from 1987 to 2000. The focus included geriatric rehabilitation and preserving function. The size of the unit averaged 10 beds during that time with an average of 2 to 4 geriatric consults per day. Approximately 50% of consults resulted in a transfer to the unit, with the remainder managed with recommendations to reduce risk of hospital acquired conditions, management of geriatric syndromes, and disposition recommendations. In 2000, the Tennessee Valley Healthcare System united the Nashville campus with the Murfreesboro campus situated 40 miles away, under one administration. In that year the intermediate care designation was withdrawn due to acute care space constraints at the Nashville Campus and the unit functioned as a mobile ACE unit without contiguous bed designation. The VA Nashville campus operated 393 acute care beds in 1991, and by 2017 was reduced to 238 beds. This was a result of a change in facility utilization with the development of expanded outpatient services F [8]. In November 2017 the GEM unit was transferred to the CLC as a four-bed unit at the Murfreesboro Campus. The GEM unit performed over 12,000 consults and cared for a total of 5380 patients at the Nashville campus over a 30-year time frame. 

An ACE unit was initiated at the San Francisco VA Medical Center in 2010 occupying 10-beds of a 20-bed geographically-based acute care specialty unit averaging approximately 50 admissions monthly, focused on preventing iatrogenic complications in high-risk, high-need, community dwelling adults 65 years of age and older admitted to the hospital for an acute illness. By design, the San Francisco VA Medical Center ACE model specifically focused on preserving/regaining function, preventing/treating delirium, and medication review. The unit aimed to have uncluttered hallways, handrails, large clocks, low beds, elevated toilet seats, and large writing boards to post information. Additionally, included was a community room where Veterans could interact with staff or other patients, eat meals together, and participate in activities that enhance mobility and cognitive abilities. The ACE service was comprised of a full-time geriatrician (MD), a full time geriatric certified nurse specialist (CNS), and shared time physical therapist (PT), occupational therapist (OT), social worker, and pharmacist. The primary team managed the acute illness and the ACE team focused on preserving function, preventing or treating delirium, and addressing polypharmacy and medication management during the hospitalization. The ACE service experienced high demand for services and in order to better meet the needs of older adults across the medical center, in 2017 it transitioned to a mobile ACE model screening all veterans admitted to the medical service age 85 years and older, regardless of location. The geriatric consult focused on management of geriatric syndromes, avoidable acute care complications, with optional transfer to the ACE Service. The San Francisco VA Medical Center has a capacity of 124 acute care beds with a closely affiliated adjacent 120-bed CLC.

The geriatric program at Tennessee Valley Healthcare System responded to an initial need to assess acute care patients remaining on acute awards with a focus on rehabilitation and disposition. As the Medical Center adopted new programs including transplants in higher acuity patients, the program focus adapted to the higher acuity rehabilitation needs of the referral population and avoidance of institutionalization. The San Francisco VA Medical Center ACE program developed an acute inpatient ward some 20 years following initiation of the Tennessee Valley Healthcare System unit, focusing on preventing iatrogenic complications of hospitalized elderly. Both locations experienced the development of geriatric services in home and outpatient settings. Long-term care community living center resources remained stable at the San Francisco VA Medical Center, however Tennessee Valley Healthcare System enhanced its skilled long-term care services with the addition of locally training geriatric personnel including pharmacists, nurse practitioners, and geriatricians, later hired by the health system and capable of providing specialized geriatric evaluation and management care.

### 2.2. Data Collection

Tennessee Valley Healthcare System GEM operations data was collected from 1987 to 2017 and included age, length of stay, disease state, disposition, and mortality data. Run chart analysis of trends was performed with descriptive and comparative statistics applied. San Francisco VA Medical Center collected length of stay, readmission rates, average daily census, and discharge to long-term-care between 2011 and 2016. Since 2017, the mobile ACE program has collected age and reason for consult request data for the geriatric consult service. Historical program development information from both sites was provided by the contributing authors. 

The Tennessee Valley Healthcare System-Institutional Review Board has designated this project as Quality Improvement.

## 3. Analysis 

The Tennessee Valley Healthcare System GEM unit had a mean patient age of 73 (SD 2.4) years, and the proportion of patients over age 65 averaged 69 (SD 8.2) % of total patients. While approximately 30% of patients with anticipated discharge to long-term care were able to return home, there was a progressive decline in the percentage of those over age 65 with time and a corresponding increase in the medically complex population less than age 65. The 30-day acute-care readmission rate remained 10% between 2015 and 2017.

**Figure 1:**
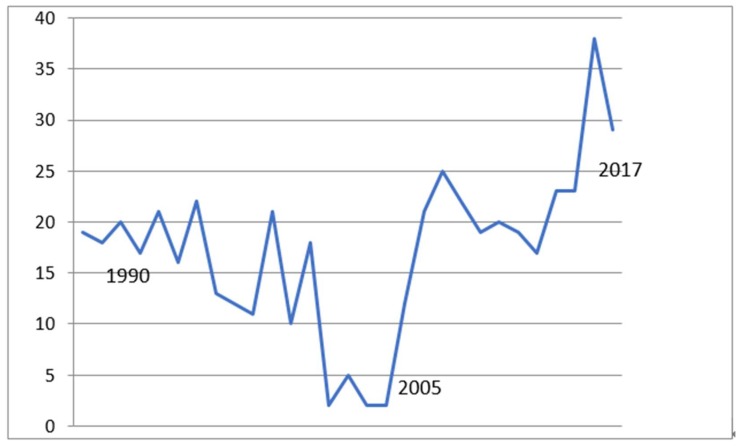
Discharge to long-term care (%). Mean % discharge to LTC (1987–2005) 13.5% (SD 3.6), (2006–2017) 22.3% (SD 4.1), F = 11.76, *p* < 0.01.

GEM functioned predominantly as a 10-bed unit, but size varied between 6 and 26 beds at different points depending on space availability and facility need. There were increased transfers to long-term care, especially 2005–2017 (Figure 1) with mean 27 (SD 13) % discharges to long-term care sent to the Murfreesboro long-term care community living center by 2015–2017. Additionally, there were increased numbers of transfers to the Murfreesboro Hospice Unit following its creation in 2000, with 38% Nashville patients occupying the 12-bed unit in 2016 to 2017 (Table 1).

**Figure 2:**
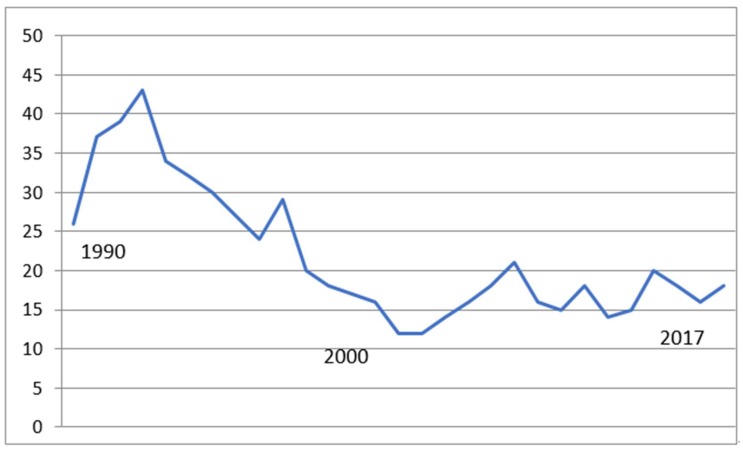
Post GEM transfer length of stay (LOS) (days). Mean LOS (1987–2000) 29.9 (SD 4.8) days, (2001–2017) 16.2 (SD 1.3) days, F = 49, *p* < 0.0001.

GEM mean length of stay (LOS) dropped dramatically from the 1990s when LOS was 29.9 days (SD 4.8) to 16.2 days (SD 1.3) from 2000 to 2017 (Figure 2) However, the length of stay was unchanged from 2000 to 2017, reflecting a constant average 16 days for these high-risk, high-need patients. The GEM population included geriatric rehabilitation and at times also homeless patients, transplant patients, dementia patients and other difficult-to-place patients depending on facility demands.

Major changes at Tennessee Valley Healthcare System over the development timeline for GEM are listed in Table 2. This included the development of primary care and outpatient services during the 1990s and a case manager program to improve patient-centered care and to facilitate discharge planning. In 1995 a Geriatric Psychiatry and in 1999 a Geriatric Medicine Fellowship program and a Geriatric Research, Education, and Clinical Center (GRECC) were established. These developments led to enhanced geriatric training for all healthcare disciplines, with eventual recruitment of eight trained geriatricians and many advanced practice nurses, clinical pharmacists, psychologists, and social workers to the clinical staff. This increase in clinical expertise permitted the development of a hospice care unit in Murfreesboro in 2000 and enhanced the ability to care for higher acuity patients in the CLC. The 2000 unification of the Nashville and Murfreesboro Campuses gradually improved coordination and increased the availability of long-term care services. Competing acute care space constraints increased at the Nashville Campus with the introduction of specialized heart failure, and bone marrow, liver, and cardiac transplant services. 

The geriatric patient centered medical home model termed geriatric patient-aligned care team, or Geri-PACT [9] was initiated in 2011 to provide enhanced outpatient geriatric evaluation, becoming widespread with over 60 Geri-PACT programs operating nationally by 2014. In 2016 the Mid-South Healthcare System, which includes the Tennessee Valley Healthcare System, mandated Geri-PACT at all facilities as part of its strategic plan. In 2015 the Strategic Analytics for Improvement and Learning Value Model (SAIL) was introduced by the Department of Veterans Affairs to measure, evaluate, and benchmark quality and efficiency internally at its medical centers. SAIL is a web-based, balanced scorecard focused on value-based population management with nine domains: Performance Measures, Patient Satisfaction, Mortality, Length of Stay, Access, Efficiency, Ambulatory Care Sensitive Conditions, Hospitalizations, and Avoidable Adverse Events [10]. The advent of SAIL and a publicly reported 5-Star rating system in 2016 heavily influenced administrative performance objectives regarding quality indicators, critical review of utilization of different levels of care, and customer satisfaction. Tennessee Valley Healthcare System received an initial 1-Star rating in 2016, and advanced to a 2-Star rating in 2018. In the fourth quarter of 2017, the Tennessee Valley Healthcare System long-term care community living center was awarded the highest 5-Star rating.

During its years of operation, the San Francisco VA Medical Center ACE unit ran an average daily census of 13 (range 6–18) veterans aged 65 and older. The LOS steadily decreased over the period of operation, from 7.9 days in 2011 to 5.3 days in 2016, consistent with national acute care trends. The 30-day readmission rate to the ACE Unit was 4.1% (national VA average 18.4% in 2012) with a discharge to a skilled nursing facility of 39.5% (national VA average in 2012 was 27%). Other geriatric syndromes or care needs were addressed as needed. The ACE unit was a primary training environment for geriatric fellows (two required rotations/year) as well as elective rotations for medical students, internal medicine residents, advanced practice nursing students, pharmacy residents and students, and therapy students. Despite efforts to triage patients appropriately between the ACE unit and other general patient units, due to the small hospital size and bed-availability, it was not unusual that the geographic ACE unit would have patients admitted in less need of geriatric services, for example meeting age but not functional criteria, while older or more frail patients who would benefit from geriatric consultation would be admitted to another unit. After initiating the mobile ACE model, automatic screening of all adults aged 85 and older was performed as close to admission as possible. The geriatrics consult service averaged 3.2 (range 0–7) new formal geriatrics consults/week with an average age of 78 (range 62–91). The most common reasons for consultation were delirium (39%), failure to thrive (18%), treatment of dementia related behaviors (15%), assistance with polypharmacy or medication management (10%), and falls (10%). Other reasons included multi-morbidity, capacity assessment, peri-operative management, help with dispositions, elder abuse, depression, and urinary retention. Many consults included requests for management of several conditions. 

Like Tennessee Valley Healthcare System, the San Francisco VA Medical Center has Home Based Primary Care, Geri-PACT clinic as well as outpatient geriatrics consult and telehealth services. In addition, the San Francisco VA Medical Center has a high-needs, intensive outpatient management consult team called IMPACT (intensive management patient aligned care team) that works with PACT providers (geri and non-geri) to assist patients in times of high medical or social need. The San Francisco VA Medical Center has a 3-Star designation, and has a limited CLC capacity.

## 4. Discussion

Both services are located in busy acute-care hospitals with active geriatric training programs and have a similar consultation rate of 2 to 4 patients per day, but developed with different foci. The San Francisco VA Medical Center provides mobile acute-care for elder patients focusing on older and frailer adults most likely to benefit from interventions, with additional consult services to younger adults as deemed needed by the primary teams. Once discharged from the acute setting, the mobile ACE team does not follow the patients into the long-term care community living enter or other skilled nursing facilities for rehabilitation or subacute care. However, Tennessee Valley Healthcare System also targeted potential rehabilitation and difficult-to-place patients, including the rehabilitation of 30% of patients with anticipated long-term care dispositions to achieve home discharge. The Tennessee Valley Healthcare System GEM program experienced a gradual increase in care for complex medical patients and a decline in mean age concurrent with an increase in medical specialty and transplant program expansion at the Nashville Campus. The benefits of GEM included a reduced 30-day acute-care readmission, improved functional status, reduced numbers of medications, and improved continuity of care for complex patients requiring specialty consultation. GEM improved the ability to address social and behavioral determinants of health and to provide continuity of inpatient care with a patient-centric discharge planning process for high-risk, high-need patients. The service also off-loaded teaching services, provided a stable interdisciplinary treatment team with consistency of staffing and program focus. It also facilitated geriatric education and research, and created a model interdisciplinary team [8]. However, the cost of GEM included interdisciplinary team personnel with required time commitments competing with other facility program needs, as well as competition for acute care beds. A 16-day GEM length of stay persisted since 2000, despite the unification of campuses. The opportunity cost for GEM included the availability of alternative care sites capable of complex care, especially the long-term care community living center and hospice at the Murfreesboro Campus, staffed by providers trained by the facility’s Geriatric Medicine and Psychiatry programs. 

Priorities favoring geriatric evaluation location in acute care include a focus in preventing acute care iatrogenic complications, medical center leadership viewing it as a priority helpful in achieving SAIL metrics, valuing the educational role of the unit, and CLC capability. While loss of a contiguous bed designation could produce challenges to maintain the geriatric environment of care (Table 3) due to lack of consistent dedicated unit staffing, it provides greater opportunity for education of staff across units resulting in “geriatricizing” non-geriatrics clinicians, including physicians, nurses, and aides. For example, it is not unusual now that the requesting consult may be a nurse on the intensive care step down unit, noticing mental status changes in a patient and implementing delirium precautions on their own while asking physicians to call a geriatrics consult for assistance.

Current VA policy emphasizes Choose Home, an initiative developed to facilitate coordination of non-institutional care to allow veterans to remain in their homes. Outpatient geriatric evaluation is available through Geri-PACT, a patient-centered medical home which provides care for frail elderly veterans by a geriatrics-trained interdisciplinary team, where more complex patients with involved medical histories can receive in-depth attention. Care is provided to geriatric patients through coordinated referral to long-term services and supports and interdisciplinary provision of medical, nursing, psychosocial, allied health services for disease treatment and prevention, health promotion and education, referral for specialty, rehabilitation and other levels of care, follow-up and overall care management by the primary care provider and support team [9].

Geriatric consultation, both inpatient and outpatient, may be another efficient means to employ scarce geriatric resources. The effectiveness of consult care could be diminished due to the limited ability to control interventions, however greater availability of patient-centered primary care at the VA (Primary Care PACT) accepting ownership of patient management across the continuum of care, combined with efficient utilization of home and community-based resources may be an effective strategy to enhance geriatric evaluation and care for patients not followed in Geri-PACT.

Home Based Primary Care (HBPC) includes health care services provided to veterans in their home. A VA physician supervises the nurse practitioner led health care team providing the services. Home Based Primary Care is for Veterans who have complex health care needs for whom routine clinic-based care is not effective, or for whom travel for office visits is not possible [11]. 

A review of program elements comprising ACE units identified three key concepts: (1) Geriatric medical review; (2) early rehabilitation; and (3) patient-centered care. These individual, focused interventions appear to have a greater effect than either discharge planning processes, or environment of care [2]. Since its inception in the 1970s, there has been an evolution of VA geriatric evaluation and management over time and resources have become more available in long-term care and outpatient settings. VA Geriatric evaluation services now comprise 34 inpatient (one mobile ACE and remainder long-term care community living center programs), 64 outpatient Geri-PACT, over 116 HBPC, and many skilled and non-skilled home and community-based services as result of the Mill Bill [11,12]. 

Geriatric services at the VA now comprise a full spectrum of care representing a significant advance from strictly inpatient geriatric evaluation. Recently, new tools to identify high-risk, high-need patients have been developed to further assist targeting higher intensity care to appropriate populations. These tools include the care assessment of need (CAN), a clinically-based predictor of future hospitalization and death developed utilizing the Primary Care Management Module in the Veterans Health Administration’s Corporate Data Warehouse [13,14,15]. Other dashboards are also under development including a specialized Geri-PACT dashboard which includes the JEN Frailty Index derived from clinical and claims-based long-term care data [16,17,18]. Facilities utilize a high-need, high-risk list to target services such as HBPC. Risk indices may be adapted for identification of other high utilizing populations applicable to oncology, heart failure, or palliative care populations for appropriate targeting of high-intensity services.

The marked variability in the reported size and length of stay of different geriatric evaluation programs suggests that program location is likely influenced by local needs and availability of workforce and resources. Previous reviews of geriatric evaluation availability and program closure or relocation suggest that the availability of interdisciplinary team members and sufficient resources for geriatric evaluation services are critical [1]. Our experience suggests that patient population characteristics, length of stay, and availability of trained personnel and alternative placements capable of meeting the needs of high-risk, high-need patients may also be important considerations. The VA guidelines appear to be adapting to the evolution of the model, including the addition of outpatient and in-home evaluation as alternative sites for geriatric evaluation programs. 

Patient selection consistent with facility priorities is important to ensure that geriatric evaluation services are placed in the optimum location and provided to appropriate patient populations. Hospital size, length of stay, and competing space obligations are important local factors to consider. Availability of resources and trained personnel across the continuum of care as well as administrative facilitation of collaboration between care components are critical to the efficient utilization of geriatric evaluation services.

## 5. Future Direction and Policy Implication

Barriers exist to maintaining acute care geriatric models, as acute care beds are at a premium in most medical centers. Facilities may need to prioritize their resources and utilize the most effective and relevant elements of geriatric evaluation and management according to the patient population, available space, resources, and institutional priorities.

## Figures and Tables

**Table 1:** Geriatric evaluation and management utilization trends, Tennessee Valley Healthcare System 1987–2017.

Decline in population over age 65
Increase in the medically complex population
Length of stay unchanged from 2000 to 2017
Increased transfers to Murfreesboro CLC
Increased transfers to Murfreesboro Hospice

**Table 2:** Developmental program milestones.

**Tennessee Valley Healthcare System Milestones**
Development of primary care and outpatient services 1990s
Development of a case manager program
1990s development of Geriatric Fellowship programs to train faculty
1999 Geriatric Research Education and Clinical Center awarded
2000 unification of campuses under one administration
2000 development of a hospice care unit, Murfreesboro Campus
Increase in transplant population and specialty services
Increased capability of the CLC since consolidation
Competing acute care space constraints, geographic IMC bed designation cessation
Geri-PACT initiated 2011, regional mandate 2016
2015 SAIL Metrics and 5-Star rating system
**San Francisco ACE Milestones**
Strong geriatric training program since 1997 In 2010 developed a shared 20-bed geographic ACE Unit
2017 mobile ACE service without geographic bed designation, seeing all patients over age 85
2017 Inpatient geriatrics consult screening for all medical patients over age 85
Small long-term care community living center capacity and bed availability
Recognition of ACE educational value and contribution to SAIL Metrics and 5-Star rating system

**Table 3:** Value-added attributes of geriatric evaluation beds.

**Benefits**
Reduced 30-day acute care readmission
Continuity of care for complex patients requiring specialty consultation
Stable interdisciplinary team, and consistency of staffing
Ability to address social behavioral determinants of health
Facilitation of geriatric education and research
**Costs**
Interdisciplinary team member cost, availability
Acute care space at a premium
Availability of alternative care sites and dispositions
Persistently prolonged length of stay for difficult-to-place patients
**Priorities Favoring Geriatric Evaluation in Acute Care**
Acute care focus on preventing iatrogenic complications, maintaining function
Medical Center leadership views as a priority
Hospital size, length of stay, space availability
Educational mission
Contribution to achieving SAIL metrics
**Priorities Favoring Geriatric Evaluation in CLC and Outpatient Locations**
Availability of space and resources in CLC, Outpatient
Availability of trained staff in CLC, Outpatient for high-risk high-need patients
Administrative commitment to close cooperation among resource units

## References

[1] Wieland D., Rubenstein L.Z., Hedrick S.C., Reuben D.B., Buchner D.M. (1994). Inpatient geriatric evaluation and management units (GEMs) in the veterans health system: Diamonds in the rough?. J. Gerontol..

[2] Fox M.T., Sidani S., Persaud M., Tregunno D., Maimets I., Brooks D., O’Brien K. (2013). Acute care for elders components of acute geriatric unit care: Systematic descriptive review. J. Am. Geriatr. Soc..

[3] Rubenstein L.Z., Josephson K.R., Wieland G.D., English P.A., Sayre J.A., Kane R.L. (1984). Effectiveness of a geriatric evaluation unit—A randomized clinical trial. N. Engl. J. Med..

[4] Landefeld C.S., Palmer R.M., Kresevec D.M., Fortinsky R.H., Kowal J. (1995). a randomized trial of care hospital medical unit especially designed to improve the functional outcomes acutely ill older patients. N. Engl. J. Med..

[5] Fox M.T., Persaud M., Maimets I., O’brien K., Brooks D., Tregunno D., Schraa E. (2012). Effectiveness of acute geriatric unit care using Acute Care for Elders components: A systematic review and meta-analysis. J. Am. Geriatr. Soc..

[6] Hung W.W., Ross J.S., Farber J., Siu A.L. (2013). Evaluation of the Mobile Acute Care of the Elderly (MACE) service. JAMA Int. Med..

[7] (2017). Department of Veterans Affairs, VHA Directive 1140.04. Geriatric Evaluation. https://www.va.gov/vhapublications/ViewPublication.asp?pub_ID=5619.

[8] Hauser B., Robinson J., Powers J.S., Laubacher M.A. (1991). The evaluation of an intermediate care–geriatric evaluation unit in a veterans administration Hospital. Southern Med. J..

[9] Department of Veterans Affairs Geri-PACT Handbook 1101.10 (1). www.va.gov/vhapublications/ViewPublication.asp?pub_ID=2977.

[10] VA Sail Metrics. http://www.va.gov/QUALITYOFCARE/measure-up/SAIL_definitions.asp.

[11] Beales J.L., Edes T. (2009). Veteran’s Affairs Home Based Primary Care. Clin. Geriatr. Med..

[12] Department of Veterans Affairs Patient Care Services. Geriatrics and Extended Care Program. https://www.patientcare.va.gov/geriatrics.asp.

[13] Care Assessment Need (CAN) Score and the Patient Care Assessment System (PCAS): Tools for Care Management. Stephan Fihn MD MPH Tami Box PhD Office of Informatics and Analytics Veterans Health Administration. https://www.hsrd.research.va.gov/for_researchers/cyber_seminars/archives/713-notes.pdf.

[14] Wong E.S., Rosland A.M., Fihn S.D., Nelson K.M. (2016). Patient-Centered Medical Home Implementation in the Veterans Health Administration and Primary Care Use: Differences by Patient Comorbidity Burden. J. Gen. Intern. Med..

[15] Wang L., Porter B., Maynard C., Evans G., Bryson C., Sun H., Gupta I., Lowy E., McDonell M., Frisbee K. (2013). Predicting risk of hospitalization or death among patients receiving primary care in the Veterans Health Administration. Med. Care.

[16] Department of Veterans Affairs Geriatrics and Extended Care Data Assessment Center (GECDAC) Measures. https://www.va.gov/geriatrics/gecdac/measures.asp.

[17] Department of Veterans Affairs Geriatrics and Extended Care Data Assessment Center (GECDAC) GeriPACT Dashboard. https://reports.vssc.med.va.gov/ReportServer/Pages/ReportViewer.aspx?%2fGeriatrics%2fGeriPACT%2fGeriPACT_Dashboard_ver2&rs:Command=Render.

[18] Kinosian BWieland D., Gu X.L., Stallard E., Phibbs C., Intrator O. (2018). Validation of the JEN Frailty Index in the National Long-Term Care Survey community population: Identifying functionally impaired older adults from claims data. BMC Health Serv. Res..

